# Polycaprolactone—Vitamin E TPGS Micellar Formulation for Oral Delivery of Paclitaxel

**DOI:** 10.3390/polym16152232

**Published:** 2024-08-05

**Authors:** Ziyad Binkhathlan, Raisuddin Ali, Osman Yusuf, Abdullah H. Alomrani, Mohamed M. Badran, Abdullah K. Alshememry, Aws Alshamsan, Faleh Alqahtani, Wajhul Qamar, Mohamed W. Attwa

**Affiliations:** 1 Department of Pharmaceutics, College of Pharmacy, King Saud University, Riyadh 11451, Saudi Arabia; ramohammad@ksu.edu.sa (R.A.); osmanhlb@yahoo.com (O.Y.); aomrani@ksu.edu.sa (A.H.A.); mbadran@ksu.edu.sa (M.M.B.); aalshememry@ksu.edu.sa (A.K.A.); aalshamsan@ksu.edu.sa (A.A.); 2 Nanobiotechnology Research Unit, College of Pharmacy, King Saud University, Riyadh 11451, Saudi Arabia; 3 Department of Pharmaceutics, Faculty of Pharmacy, Al-Neelain University, Khartoum 11121, Sudan; 4 Department of Pharmacology and Toxicology, College of Pharmacy, King Saud University, Riyadh 11451, Saudi Arabia; afaleh@ksu.edu.sa (F.A.); wqidris@ksu.edu.sa (W.Q.); 5 Department of Pharmaceutical Chemistry, College of Pharmacy, King Saud University, Riyadh 11451, Saudi Arabia; mzeidan@ksu.edu.sa

**Keywords:** block copolymer, polycaprolactone–vitamin E TPGS, polymeric micelles, paclitaxel, oral, intestinal permeability, pharmacokinetics

## Abstract

This study aimed to investigate the potential of polycaprolactone–vitamin E TPGS (PCL-TPGS) micelles as a delivery system for oral administration of paclitaxel (PTX). The PCL-TPGS copolymer was synthesized using ring opening polymerization, and PTX-loaded PCL-TPGS micelles (PTX micelles) were prepared via a co-solvent evaporation method. Characterization of these micelles included measurements of size, polydispersity, and encapsulation efficiency. The cellular uptake of PTX micelles was evaluated in *Caco-2* cells using rhodamine 123 (Rh123) as a fluorescent probe. Moreover, an everted rat sac study was conducted to evaluate the ex vivo permeability of PTX micelles. Additionally, a comparative pharmacokinetic study of PTX micelles versus the marketed formulation, Ebetaxel^®^ (a Taxol generic), was performed after a single oral administration to rats. The results demonstrated that the micellar formulation significantly improved PTX solubility (nearly 1 mg/mL). The in vitro stability and release of PTX micelles in simulated gastric fluid (SGF) and simulated intestinal fluid (SIF) demonstrated that PTX micelles remained stable for up to 24 h and significantly slowed the release of PTX in both media compared to Ebetaxel^®^. The in vitro cellular uptake, ex vivo intestinal permeability, and in vivo pharmacokinetic profile demonstrated that PTX micelles enhanced the permeability and facilitated a rapid absorption of the drug. Conclusively, the PCL_7000_-TPGS_3500_ micelles exhibit potential as an effective oral delivery system for PTX.

## 1. Introduction

Chemotherapy is considered a mainstay in the treatment of several types of cancer. Unfortunately, however, most anticancer drugs cannot be taken orally due to their limited water solubility and/or poor permeability in the gastrointestinal (GI) tract. Therefore, in the current practice, anticancer agents are administered via intravenous (IV) injection. This method is not without drawbacks, as it inconveniences patients and places a financial burden on the healthcare system. Additionally, IV administration of anticancer drugs often leads to severe side effects, either from the drugs themselves or occasionally from the added excipients.

Paclitaxel (PTX) is a potent anticancer drug widely employed to treat various cancer types, such as ovarian, breast, endometrial, and non-small cell lung cancers, and AIDS-related Kaposi’s sarcoma [1,2,3,4,5,6,7]. According to the Biopharmaceutics Classification System (BCS), PTX is considered as a Class IV drug due to its low solubility and permeability characteristics. It exhibits high lipophilicity, indicated by a LogP value of 3.66, and is practically insoluble in water (~0.3 μg/mL). The reported pKa value for PTX is 10.36, suggesting a very weak acidic nature of the drug [8]. The conventional formulation of PTX comprises a combination of 50% Kolliphor^®^ EL and 50% anhydrous ethanol, commercially known as Taxol^®^. Apart from Taxol, there are three other PTX products currently available in the market, including albumin-bound PTX (Abraxane^®^), methoxy polyethylene oxide-b-poly D,L-lactide micellar formulation (Genexol^®^-PM/Cynviloq^TM^), and PTX micellar (Apealea^®^/Paclical^®^), which is composed of a mixture of micelle-forming retinoic acid derivatives [9]. The administration of all these products is limited to the IV route only.

Currently, there are no approved oral formulations of PTX for clinical use. It has been shown that Taxol exhibits very low oral bioavailability in humans (4%) [10]. It is believed that P-glycoprotein (P-gp) restricts the transport of PTX from the GI tract, leading to the drug’s low permeability in the GI tract and consequently its low bioavailability. This notion is supported by a study conducted on *Mdr1a* knockout mice by Sparreboom et al. [11], where they investigated the pharmacokinetics of PTX in wild-type and *Mdr1a* P-gp knockout mice after oral and IV administration. After oral administration, *Mdr1a* knockout mice showed a more than sixfold increase in the plasma area under the concentration–time curve (AUC) of PTX compared to wild-type mice. Similarly, following IV administration, *Mdr1a* knockout mice displayed a twofold increase in the plasma AUC of PTX compared to wild-type mice. The results from drug excretion studies led to the conclusion that intestinal P-gp not only reduces the bioavailability of orally administered PTX but also contributes to the drug’s elimination following IV administration by directly secreting PTX into the intestinal lumen [11]. Based on these findings, attempts have been made to improve the low and variable oral bioavailability of PTX by co-administering P-gp inhibitors. Numerous clinical trials have been conducted to evaluate the feasibility and safety of this approach, using P-gp inhibitors such as cyclosporine A (CyA) or HM30181A, a novel oral P-gp inhibitor [12,13,14,15,16].

However, it should be noted that some of these P-gp/cytochrome P-450 (CYP-450) inhibitors, like CyA, have immunosuppressive effects, which may lead to clinical complications. Additionally, these inhibitors have their own adverse effects and challenges in formulating them for clinical use [17]. To successfully formulate oral chemotherapy, the development of specific and less toxic inhibitors would be a crucial step.

D–α–tocopheryl polyethylene glycol succinate (TPGS) has gained significant prominence in the pharmaceutical industry in recent times. It offers a wide range of applications serving as an absorption enhancer, emulsifier, solubilizer, additive, and stabilizer. Moreover, TPGS has been utilized as a P-gp inhibitor to improve the oral bioavailability of P-gp substrates, including PTX [18]. Additionally, TPGS has been employed in prodrug design to enhance chemotherapy outcomes.

Lately, a series of copolymers comprising polycaprolactone (PCL) and TPGS was effectively developed and characterized in our laboratory. We showed that through a co-solvent evaporation method, the optimized PTX-loaded PCL-TPGS micelles efficiently encapsulate PTX. Particularly, PCL_7000_-TPGS_3500_ micellar formulation significantly improved the PTX solubility to achieve a clinically relevant aqueous solubility of nearly 1 mg/mL. Additionally, these micelles exhibited a considerably slower release of PTX in vitro compared to the commercially available PTX formulation, Ebetaxel^®^ (a generic version of Taxol^®^) [19]. Further, the in vitro cytotoxicity of the developed PCL_7000_-TPGS_3500_ micellar formulation was comparable to Ebetaxel^®^.

The primary aim of this study was to investigate the suitability of PCL_7000_-TPGS_3500_ micelles as a delivery system for oral administration of PTX. We examined the stability of PTX-loaded PCL_7000_-TPGS_3500_ micelles (PTX micelles) in simulated gastric and intestinal fluids. The in vitro drug release from PTX micelles was also evaluated and compared to Ebetaxel^®^ in both media. Moreover, an everted rat sac study was conducted to evaluate the ex vivo permeability of PTX micelles. Additionally, the cellular uptake of rhodamine 123-loaded PCL-TPGS micelles was studied in *Caco-2* cells. We finally performed a comparative pharmacokinetic study of PTX micelles versus the marketed formulation, Ebetaxel^®^, following a single oral administration to rats.

## 2. Materials and Methods

### 2.1. Materials

TPGS_3500_ was sourced from Wuhan Jason Biotech Co, Ltd. in Wuhan, Hubei, China. D–α–tocopherol polyethylene glycol 1000 succinate (TPGS_1000_), methoxy polyethylene oxide (Mn 5000), stannous octoate at a purity of approximately 95%, rhodamine 123 (Rh123), ε-caprolactone with a purity of 97%, and HPLC-grade THF were acquired from Sigma-Aldrich, based in St. Louis, MO, USA. Deuterated chloroform (CDCl_3_) with a purity of 99.8% was purchased from Cambridge Isotope Laboratories Inc. located in Tewksbury, MA, USA. Sodium chloride injection (USP) at 0.9% concentration was procured from Pharmaceutical Solutions Industry located in Jeddah, Saudi Arabia. PTX with a purity of 99% was obtained from Shaanxi Sciphar Biotechnology Co., Ltd. based in Xianyang, Shijiyiuan, China. Ebetaxel^®^ (300 mg/50 mL), an approved generic version of Taxol^®^, was acquired from King Khalid University Hospital Pharmacy in Riyadh, Saudi Arabia, Lot# HN5538. Simulated gastric and intestinal fluids (SGF and SIF) were bought from Biorelevant.com Ltd. in London, UK, and were prepared according to provided instructions. Dialysis bags (Spectra Por^®^ S/P 3 Dialysis Membrane Trial Kit, MWCO = 3.5 and 12–14 kDa, 18 mm) were acquired from Cole-Parmer Canada based in Montreal, QC, Canada. Cyclosporine A (CyA) was obtained from Molekula Limited (Newcastle, UK). Acetonitrile, ammonium acetate, methanol, and water, all of HPLC grade, were obtained from BDH Chemical Ltd. in Poole, England. All other chemicals used were of reagent grade. Deionized water was produced in-house using a Millipore system.

### 2.2. Methods

#### 2.2.1. Synthesis of PCL-TPGS Copolymer

PCL-TPGS block copolymer was synthesized by ring opening polymerization of ε-caprolactone using TPGS_3500_ as an initiator and stannous octoate as a catalyst, as previously reported [19]. Briefly, TPGS_3500_, ε-caprolactone, and stannous octoate were added to a previously flamed ampoule, nitrogen purged, then sealed under vacuum. The reaction proceeded at 140 °C for 4 h.

^1^H NMR was used to determine the molecular weight of the synthesized copolymer. ^1^H NMR spectra were analyzed using a Bruker Ultra shield 500.133 MHz spectrometer (Bruker BioSpin AG, Fällanden, Switzerland) with CDCl_3_ as the solvent. In order to ascertain the degree of polymerization of ε-CL in the copolymer, the peak intensity ratio of the methylene protons of the PCL segment (–O–CH_2_) and the methylene protons of the PEG segment (–O–CH_2_–CH_2_) was examined at δ = 4.07 ppm and 3.65 ppm, respectively [19].

#### 2.2.2. Preparation of PTX Micelles

The co-solvent evaporation method was employed to prepare PTX micelles [19]. In a typical experiment, the drug (2 mg) and PCL_7000_-TPGS_3500_ copolymer (60 mg) were dissolved in THF (1 mL) and added drop by drop into distilled water (2 mL) while stirring. Subsequently, the mixture was left to evaporate overnight at room temperature under vacuum to eliminate any residual organic solvent.

The hydrodynamic diameter (size) and PI of PTX micelles were measured using Zetasizer NanoZS (Malvern Instrument Ltd., Malvern, UK) instrument. Deionized water was used as the dispersion medium for all prepared micelles. Prior to analysis, the samples were equilibrated for 2 min at 25 °C. The measurements were performed using a non-invasive backscatter technique at 173° of detection angle. All measurements were performed in triplicate. 

To determine the drug encapsulation efficiency, 1 mL of the freshly prepared micellar dispersion was centrifuged at 13,000 rpm for 5 min to remove undissolved drug or large particles. Then, a 100 µL of the supernatant was diluted with 900 µL of acetonitrile to disrupt the micelles. The levels of encapsulated PTX were determined using a previously published HPLC-UV method [19,20]. The reported validation data for this method include “linearity r^2^: 0.9999, repeatability CV%: 0.4%, reproducibility CV%: 1.1%, LOD: 0.028 µg/mL, LOQ: 0.095 µg/mL”. The HPLC system used in the current study consisted of a Waters^TM^ Model 1515 HPLC pump, Waters^TM^ Autosampler Model 717 plus, and Waters^TM^ 2487 dual absorbance UV detector (Milford, MA, USA), all controlled by Breeze 2 software (BuildNumber: 2154; Database Version Number: 6.20.00.00). An analytical column with a C_18_ stationary phase (Sunfire^TM^; 250 mm length × 4.6 mm id, 5 µm particle size) was employed for isocratic elution. The mobile phase consisted of a mixture of acetonitrile and water (70:30 ratio), flowing at a rate of 1 mL/min, and the detection wavelength was set at 227 nm. A 30 µL injection volume was used for sample analysis. The concentration range used in the calibration curve was (0.5–100 µg/mL). The percentage encapsulation efficiency (% EE) was estimated by using the following equation:

$$\mathrm{EE}\;(\%)=\frac{\mathrm{Amount}\;\mathrm{of}\;\mathrm{loaded}\;\mathrm{PTX}\;(\mathrm{mg})}{\mathrm{Amount}\;\mathrm{of}\;\mathrm{PTX}\;\mathrm{added}\;(\mathrm{mg})}\times 100$$

#### 2.2.3. In Vitro Release of PTX from Micelles in Simulated Gastric Fluid (SGF) and Simulated Intestinal Fluid (SIF)

The release study was conducted following a method previously reported for ritonavir nanoparticles, with minor modification [21]. In summary, PTX micelles (1 mL) containing 100 µg of PTX were placed inside a dialysis bag with a molecular weight cutoff of 14 kDa. The dialysis bag was then submerged in a flask containing 50 mL of SGF with 0.5% (*v*/*v*) polysorbate 80, creating sink conditions, and shaken at 150 rpm at 37 °C for 2 h. Then, certain volumes of 2 M KH_2_PO_4_ and 0.5 N NaOH were added to adjust the pH value to 6.8 (SIF), and the study was continued for another 22 h. At predetermined time points, sample aliquots were collected. At specific time intervals, 1 mL aliquots of the release medium were collected and immediately replaced with the same volume of fresh release medium. The level of released PTX in each sample was measured using the HPLC-UV method described above.

A model-independent method, employing the difference factor and similarity factor, was utilized to compare the release patterns of PTX from Ebetaxel with PTX-loaded PCL-TPGS micelles [22]. The difference factor (*f*_1_) was computed as the percentage error between the two curves across all time points, and it is calculated as follows:


$$f_{1}=\frac{\Sigma_{j=1}^{n}|R_{j}-T_{j}|}{\Sigma_{j=1}^{n}R_{j}}\times 100$$

In the above formula, in this calculation, *n* represents the sampling number, *R_j_* stands for the percentage of PTX released from Ebetaxel^®^ (reference) at time *j*, and *T_j_* represents the percentage of PTX released from PTX-loaded micelles (test) at the same time point. The similarity factor (*f*_2_) was determined using the following equation:


$$f_{2}=50\times log\{{\left[1+(1/n)\overset{n}{\underset{j=1}{\Sigma }}{\left(R_{j}-T_{j}\right)}^{2}\right]}^{-\frac{1}{2}}\times 100\}$$

Typically, *f*_1_ values below 15 (ranging from 0 to 15) and *f*_2_ values above 50 (ranging from 50 to 100) indicate a similar dissolution (release) profile [23].

#### 2.2.4. Stability of PTX Micelles in SGF and SIF

The stability of PTX micelles was assessed for the size and PI changes upon storage in SGF and SIF conditions. Eight milliliters of PTX micelles were placed inside a dialysis bag with a molecular weight cutoff of 3.5 kDa. The dialysis bag was then immersed in a flask containing 50 mL of SGF with 0.5% (*v*/*v*) polysorbate 80. The flask was placed in a constant temperature water bath at 37 °C and shaken at 150 rpm for 2 h. Then, certain volumes of 2 M KH_2_PO_4_ and 0.5 N NaOH were added to adjust the pH value to 6.8 (SIF), and the study was continued for another 22 h. At specific time intervals, 0.2 mL aliquots were collected from inside the bag. The diameter and PI of each sample were measured using DLS.

#### 2.2.5. In Vitro Cellular Uptake Study

Rh123, a known substrate for P-gp, was used as a fluorescent probe to assess cellular uptake in *Caco-2* cell lines. *Caco-2* cells were seeded in three flat-bottom six-well plates at a density of 2 × 10^5^ cells per well. The wells were assigned to eight groups as follows: 

Group I: Control (received no treatment only fresh media without Rh123);

Group II: Free Rh123 20 µM (Rh123);

Group III: Rh123 (20 µM) encapsulated in PCL_7000_-TPGS_3500_ micelles;

Group IV: Rh123 (20 µM) in Ebetaxel^®^ vehicle (mixture of Kolliphor^®^ EL and ethanol);

Group V: Rh123 (20 µM) encapsulated in PCL_7000_-PEO_4000_ micelles;

Group VI: Solution mixture of Rh123 (20 µM) and TPGS_1000_ (33 µM);

Group VII: Solution mixture of Rh123 (20 µM) and TPGS_3500_ (33 µM);

Group VIII: Solution mixture of Rh123 (20 µM) and CyA (4 µM).

After 24 h, the cell culture medium was replaced with fresh medium containing Rh123 (20 µM), either free or encapsulated according to the assigned wells. After 5 h of incubation, the medium was carefully removed, and the cell layers washed three times with sterile PBS. The cells were then observed under a fluorescent microscope (OPTIKA^®^ Microscopes, Ponteranica, Italy). The fluorescence intensities of the images were quantified using the ImageJ software (version 1.54d, National Institute of Health, Bethesda, MD, USA) [24].

#### 2.2.6. Ex Vivo Intestinal Permeability Study in Rats

Ex vivo intestinal permeability of the optimum PTX-loaded micelles was performed using excised intestine technique. The study of the intestinal permeability of PTX formulations was performed on non-everted and everted gut sac [25]. 

Healthy rats (200–250 g) were euthanized using ketamine followed by cervical dislocation. The abdomen of each rat was opened, and the jejunum part of the small intestine was quickly removed, cut into small segments of 5 ± 1 cm length, washed with Krebs solution to remove any debris, and then immersed immediately into Krebs solution (pH 7.0). For the non-everted gut sac test, the intestinal segments were used as is, whereas in the case of the everted gut sac test, the intestinal segments were everted by turning their insides out. 

One side of each segment was firmly tied with a surgical suture and filled with 0.5 mL of the formulation containing 100 µg of PTX. Next, the other side of the intestinal segment was firmly tied with the surgical suture to prevent leaking, washed with water to remove any traces of PTX formulation, and immersed in a glass tube containing 10 mL oxygenated Krebs solution. The tubes were placed in a water bath adjusted at 37 °C with continuous shaking (50 rpm). One mL from each tube was withdrawn at different time intervals: 1, 2, 3, and 4 h. The cumulative amount of PTX permeated through the intestinal membrane was measured and different parameters, including the apparent permeability [26,27] and transport rate (flux) [27,28], of PTX micelles and Ebetaxel^®^ were calculated. The apparent permeability (*P_app_*) and Flux (*J*) were obtained using the following equations:

$$P_{app}=\;\;\frac{dQ/dt\;}{A*C_{0}}$$

$$Flux\;(J)=\;\;\frac{Q\;}{t*A}$$

*P_app_*: apparent permeability (cm/sec);

*Q*: cumulative PTX permeated (µg);

*t*: time (sec);

*A*: mucosal surface area (cm^2^);

*C*_0_: initial concentration (µg/cm^3^).

#### 2.2.7. Pharmacokinetics and Tissue Distribution Study in Rats

Animal studies were carried out following protocols approved by the Research Ethics Committee (REC) at King Saud University (Reference No. KSU-SE-22-49). Male Wistar rats weighing between 250 and 300 g were kept in temperature-controlled rooms with a 12 h light–dark cycle. Before the experiments, the animals had unrestricted access to food and water. However, food was withheld overnight prior to the study. On the day of experimentation, the rats were divided into two groups (4 rats per group): the control group received Ebetaxel^®^ solution (50 mg/mL), after a 10-time dilution with normal saline, and the test group received PTX in the PCL_7000_-TPGS_3500_ micellar formulation. Each rat in both groups was orally administered a single dose of 10 mg/kg of PTX through gavage. 

At specific time points (0.5, 1, 2, 3, 4, 8, 12, and 24 h) after oral administration, whole blood samples (100–200 µL) were obtained from each rat via the retro orbital venous plexus. At the 24th h-post dose, the animals were euthanized by cardiac puncture under deep anesthesia, and blood, liver, spleen, kidneys, heart, and lungs were collected. Whole blood samples from each rat were collected into heparinized tubes and were immediately centrifuged for 10 min to collect plasma, which were also kept at −20 °C until assessed for drug concentration. Tissue samples were washed in ice-cold saline, blotted with paper towel, weighed, and stored in phosphate buffer (pH = 7.4) at −80 °C until being analyzed. The PTX concentrations in plasma and tissue samples were analyzed using an ultraperformance liquid chromatography–mass spectrometry (UPLC–MS/MS) method, as described below, and the resulting concentration versus time curves were then profiled.

#### 2.2.8. Determination of PTX Levels in Plasma and Tissues

The plasma and tissue concentrations of PTX were determined using a UPLC-MS/MS assay method, with docetaxel as the internal standard (IS). Briefly, both PTX and docetaxel were eluted on a C_18_ column (3.5 µm, 2.1 × 150 mm), with an Acquity^TM^ column In-Line filter (0.2 µm, 2.1 mm) used to safeguard the analytical column. The column temperature was maintained at room temperature, and the mobile phase consisted of acetonitrile and water containing 0.1% formic acid in an 80:20 ratio, respectively, flowing at a rate of 300 µL/min. The autosampler temperature was kept at 20 ± 5 °C, and the injection volume was 5 µL, with a sample run time of 3.0 min.

The quantification of PTX and the IS were conducted using a triple quadrupole mass spectrometer (TQD) in positive electrospray ionization (ESI+) mode with multiple reaction monitoring (MRM) analysis. The selected MRM for PTX were 854.36 → 104.96 (cone voltage 12 V, collision voltage 56 V) and 854.36 → 286.06 (cone voltage 12 V, collision voltage 12 V). Similarly, the selected MRM for IS was 808.37 → 181.99 (cone voltage 10 V, collision voltage 34 V) and 808.37 → 226.07 (cone voltage 10 V, collision voltage 16 V). The source temperature and desolvation temperature were set at 150 °C and 350 °C, respectively, with desolvation gas (nitrogen) and cone gas set at 650 L/h and 1 L/h, respectively. The collision gas (argon) flow was set at 0.15 mL/min.

For sample preparation, frozen plasma samples were thawed at room temperature (23 ± 2 °C) and vortexed for approximately 10–15 s. A 100 µL aliquot of each plasma sample was transferred to Eppendorf tubes. Each sample was mixed with 10 µL of internal standard (IS) (100 µg/mL) and 50 µL of acetonitrile, followed by vortex-mixing for 30 s. Subsequently, 1 mL of tertiary butyl methyl ether (t-BME) was added, and the samples were vortex-mixed again for 30 s. The samples were then centrifuged at 10,000 rpm for 10 min at 4 °C. The supernatant (950 µL) from each sample was separated and dried using a vacuum centrifuge. The dried residue was reconstituted with 100 µL of acetonitrile, and 7.5 µL of each sample was injected into the LC-MS/MS. PTX calibration samples were freshly prepared by spiking plasma (100 µL each) to cover a concentration range of 10 to 2000 ng/mL

The tissues were frozen at −80 °C for two days following collection. After 2 days of storage, the tissues were allowed to thaw at room temperature and then gently patted dry with tissue towels. Immediately after drying, the tissues were accurately weighed, chopped into small pieces, and transferred into 15 mL round-bottom tubes. Then, a calculated volume of milli-Q water (300 µL of milli-Q water for each 100 mg tissue) was added to the tubes and homogenized using a T18 digital Ultra Turrax homogenizer (IKA-Werke, Staufen im Breisgau, Germany) running at a speed of 10,000 rpm. The obtained tissue suspensions were clearly labeled and stored in −80 °C prior to use. On the day of analysis, the tissue suspensions were allowed to thaw at room temperature and then vortexed for 30 s. A 300 µL aliquot of the vortexed tissue suspension was transferred into an Eppendorf tube and treated in the same manner as the plasma samples. After reconstitution, the samples were analyzed by the same UPLC-MS/MS method.

#### 2.2.9. Data and Statistical Analysis

All data are reported as mean ± SD, unless otherwise indicated. Differences between the mean values were compared using analysis of variance (ANOVA) followed by post-hoc analyses using Tukey–Kramer multiple comparison test. The pharmacokinetic parameters were calculated using the non-compartmental analysis method in PKSolver 2.0 software. The level of significance was set at α = 0.05.

## 3. Results

### 3.1. Characteristics of PCL-TPGS Copolymers and PTX Micelles

PCL_7000_-TPGS_3500_ copolymer was synthesized successfully with a total MW of approximately 10,400 g/mol. The encapsulation efficiency of PTX was approximately 93% reaching an aqueous solubility of 0.93 mg/mL in PCL-TPGS micelles (Table 1). The mean size of PTX micelles was around 68 nm (Table 1). The values for encapsulation efficiency and diameter of the PTX micelles were very similar to those we reported recently [29].

### 3.2. In Vitro Release Profile of PTX Micelles in SGF and SIF

**Figure 1:**
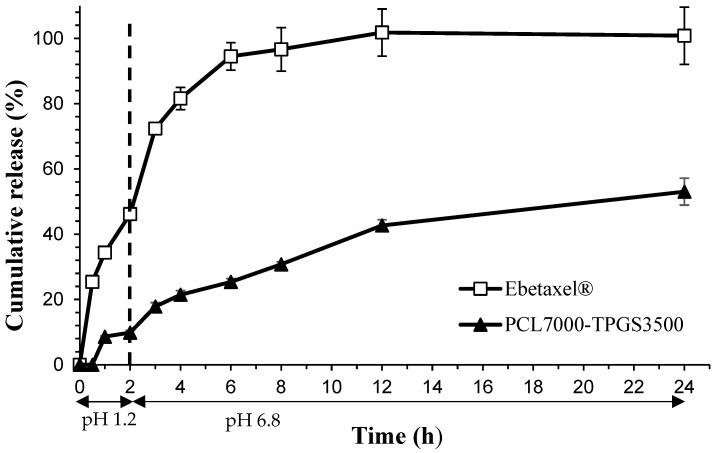
PTX release profile from PTX micelles and Ebetaxel in SGF and SIF, maintained at 37 °C and 50 rpm. Each data point represents the mean ± SD (n = 3).

Figure 1 depicts the PTX release patterns from PTX micelles and commercial formulation (Ebetaxel) in SGF and SIF. In the case of PTX micelles, the release rate of PTX was slower in both environments, with approximately 10% and 53% of the drug being released within after 2 h and 24 h incubation, respectively. In contrast, the drug release percentages obtained with Ebetaxel at these time intervals were 46.2% and 100%, respectively. 

The similarity factors *f*_1_ and *f*_2_ calculated for PTX-loaded micelles were 67.9 and 14.3, respectively. Thus, the release profile of PTX from PCL-TPGS micelles is deemed dissimilar to that of Ebetaxel.

### 3.3. In Vitro Stability of PTX Micelles in SGF and SIF

**Figure 2:**
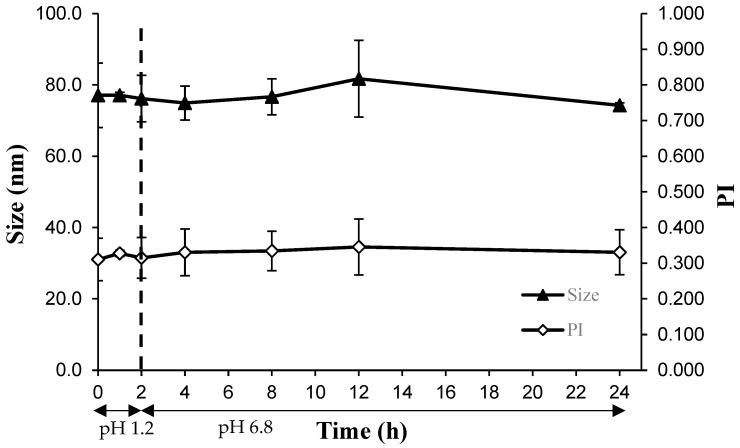
Changes in the diameter and PI of PTX micelles over time during incubation in SGF and SIF at 37 °C and 50 rpm. Each data point is presented as the mean ± SD (n = 3).

This study measured the diameter of micelles in SGF and SIF at 37 °C at various time points. Figure 2 illustrates that the Z_ave_ diameter of the micelles maintained at 74–81 nm for the entire duration of the experiment. Moreover, the PI values were essentially unchanged (0.031–0.034) throughout the 24 h incubation time (Figure 2).

### 3.4. In Vitro Caco-2 Cellular Uptake

**Figure 3:**
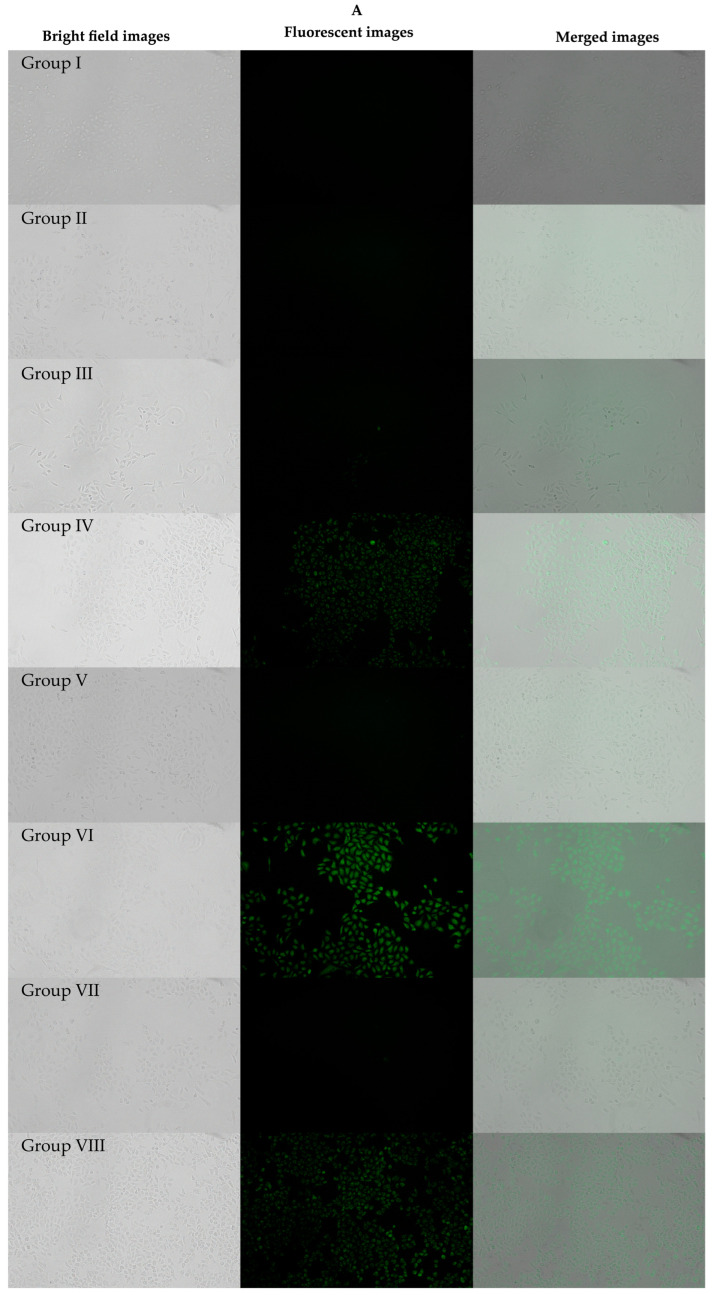

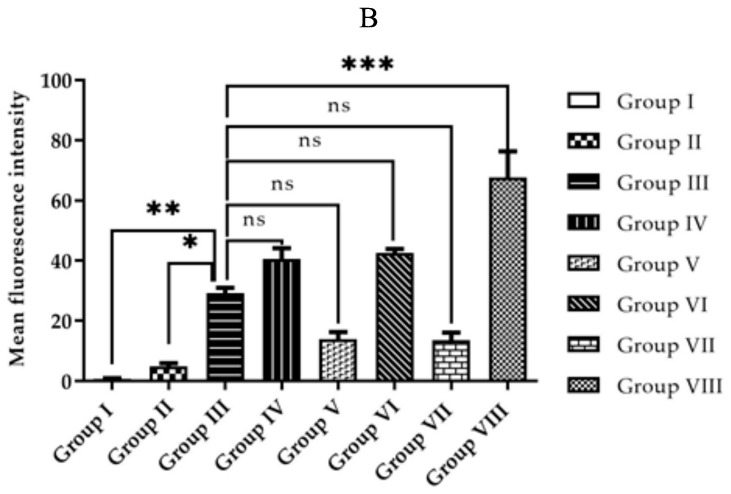
In vitro cellular uptake of Rh123 in *Caco-2* cells after 5 h of incubation at 37 °C. Bright field and green fluorescence images after incubation with different treatment groups (**A**) and comparative mean fluorescence intensity amongst various treatment groups (**B**). For a better view of the images, readers are referred to the Supplementary Material (Figure S1). Group I: control (no Rh123 treatment); Group II: free Rh123; Group III: Rh123 in PCL_7000_-TPGS_3500_ micelles; Group IV: Rh123 in Ebetaxel vehicle (Kolliphor EL + ethanol); Group V: Rh123 in PCL_7000_-PEO_4000_; Group VI: Rh123 in TPGS_1000_ solution; Group VII: Rh123 in TPGS_3500_ solution; Group VIII: Rh123 + CyA. * *p* < 0.05; ** *p* < 0.01; *** *p* < 0.005; ns: non-significant (*p* > 0.05).

In the cellular uptake study conducted on Caco-2 cells using Rh123 as a fluorescent probe, we observed varying levels of cellular uptake across the different treatment groups (Figure 3). Group I (negative control) exhibited negligible fluorescence, as expected, since no Rh123 was present. Group II, which received free Rh123, showed very low fluorescence intensity, indicating minimal cellular uptake. 

Groups III and V, which involved Rh123 encapsulated in PCL_7000_-TPGS_3500_ micelles and PCL_7000_-PEO_4000_ micelles, respectively, displayed distinct cellular uptake profiles. Group III showed a marked enhancement in fluorescence intensity compared to the free Rh123 group, suggesting that the PCL_7000_-TPGS_3500_ micelles effectively increased the permeability of Rh123. On the other hand, Group V showed a less pronounced increase, implying that the PCL_7000_-PEO_4000_ micelles were less effective at facilitating Rh123 uptake suggesting the role of TPGS as an absorption enhancer/P-gp inhibitor.

Group IV, treated with Rh123 in the Ebetaxel vehicle, presented a mean fluorescence intensity significantly higher than that of Group II (free Rh123), indicating that the presence of Kolliphor EL, which is a known P-gp inhibitor, significantly enhanced the cellular uptake of Rh123 (*p* < 0.05; ANOVA followed by Tukey–Kramer post-hoc analysis). Although the mean fluorescence intensity of Group IV was also higher than that obtained with Group III, the difference was statistically insignificant (*p* > 0.05; ANOVA followed by Tukey–Kramer post-hoc analysis). 

Group VI included Rh123 in the presence of TPGS_1000_—which is a well-known absorption enhancer/P-gp inhibitor—and demonstrated a statistically significant increase in mean fluorescence intensity compared to free Rh123 (Group II). Although Group VII—which included Rh123 in the presence of TPGS_3500_—showed a higher fluorescence intensity compared to Group II (free Rh123), it was less pronounced than that obtained with Group VI. 

Finally, Group VIII (positive control), treated with a solution of Rh123 co-incubated with cyclosporine A, exhibited the highest fluorescence intensity among all groups. This result implies that cyclosporine A, a known inhibitor of P-gp, significantly increased the cellular uptake of Rh123 by inhibiting its efflux (Figure 3).

### 3.5. Ex Vivo Intestinal Permeability of PTX Micelles

**Figure 4:**
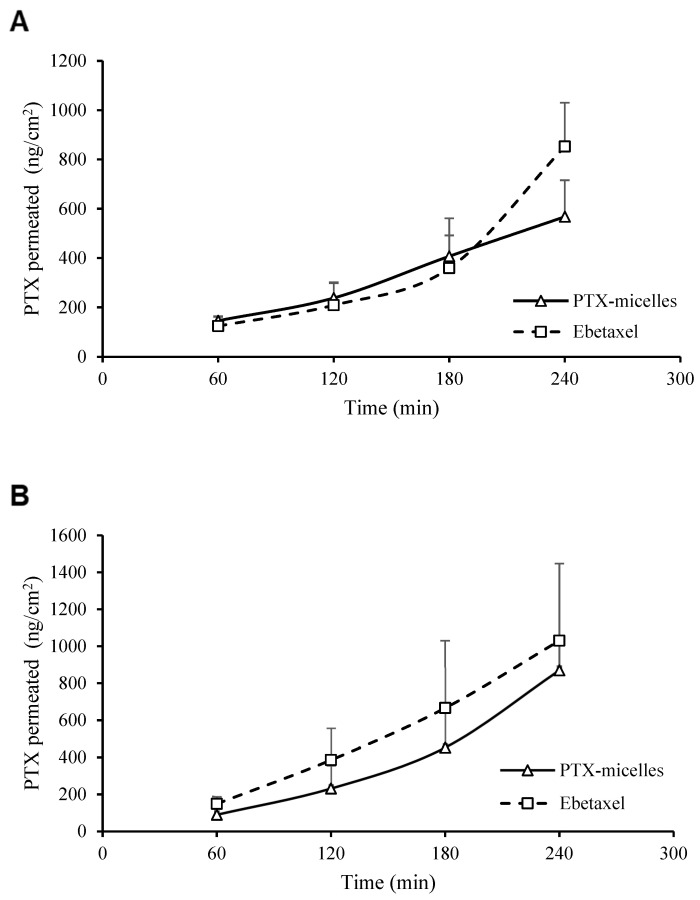
PTX permeated through non-everted (**A**) and everted (**B**) gut sacs.

The ex vivo permeation data of PTX through excised intestinal segments (non-everted and everted sacs) are presented in Figure 4 and Table 2. The rate of PTX permeated through non-everted sac from PTX-loaded micelles at the first three hours of incubation was apparently (but statistically non-significant) higher than that of Ebetaxel. However, after 4 h, the rate of Ebetaxel permeation exceeded that observed with PTX micelles (*p* < 0.05; Student’s *t*-test) (Figure 4A). One the other hand, the permeation of Ebetaxel through the everted sac was apparently (but statistically non-significantly) higher than that of the PTX micelles (Figure 4B). 

The *P_app_* values of PTX from PTX micelles and Ebetaxel through everted and non-everted gut sacs are presented in Table 2. The *P_app_* of PTX micelles through everted gut sac was higher compared to non-everted one with a ratio of 2:1. The *P_app_* ratio of everted to non-everted values for Ebetaxel decreased to (1.2:1 ratio), which could indicate the P-gp-inhibitory effect of the Ebetaxel vehicle (Kolliphor EL, formerly known as Cremophor EL). The *P_app_* value of Ebetaxel through non-everted gut sacs was higher than that of PTX micelles; however, the *P_app_* values of these formulations were comparable through everted gut sacs (Table 2).

### 3.6. Pharmacokinetics and Tissue Distribution of PTX Micelles

**Figure 5:**
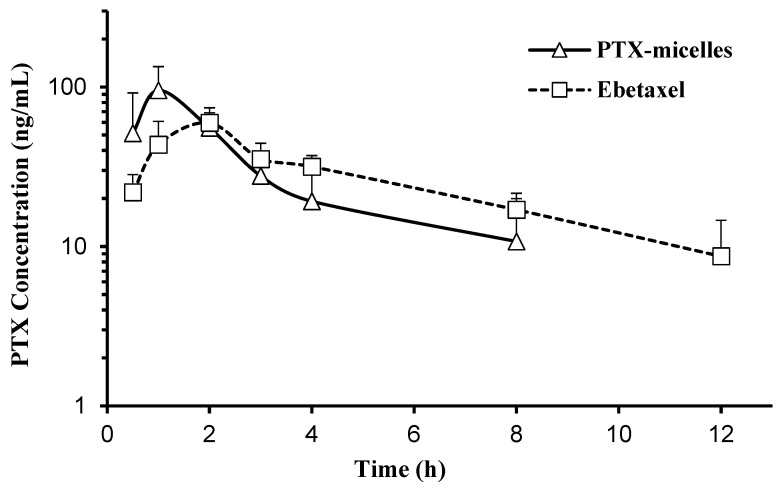
Plasma concentration–time profile of PTX following an oral dose (10 mg/kg) of Ebetaxel or PTX micelles to rats. Each data point represents the mean ± SD (n = 4 rats/group).

Figure 5 shows the plasma concentration–time curve of PTX following oral administration (10 mg/kg) of PTX micelles or Ebetaxel to rats. The pharmacokinetic profile of PTX micelles displayed a rapid absorption phase for PTX reaching a mean Cmax of 96.62 (±33.74) ng/mL, which was achieved within 1 h (T_max_). On the other hand, Ebetaxel showed a relatively slower absorption phase (T_max_ = 2 h) with a mean C_max_ of 59.60 (±8.17) ng/mL. Although the PTX micelles showed a higher peak concentration compared to Ebetaxel, the difference was statistically non-significant (*p* > 0.05). The relative bioavailability of PTX in the PTX micelles against Ebetaxel was around 84.5%. The average oral clearance rates (CL/F) were 39.23 (±14.64) L/h/kg for PTX micelles and 28.40 (±7.25) L/h/kg for Ebetaxel. The pharmacokinetic parameters of PTX are listed in Table 3. 

**Figure 6:**
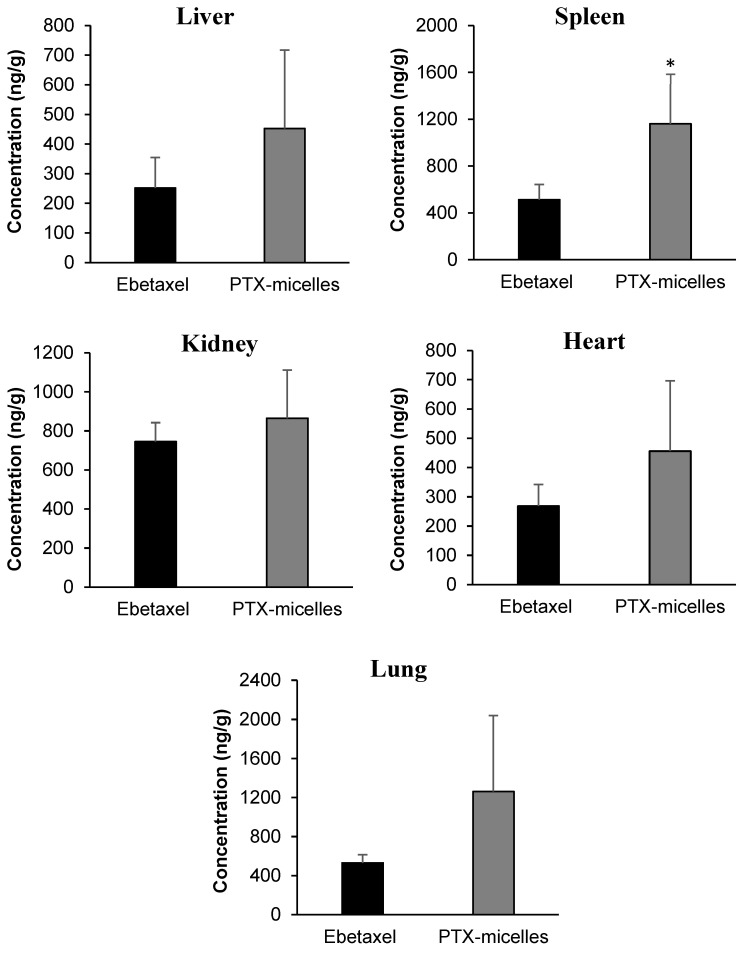
Tissue concentrations of PTX 24 h post-dose (10 mg/kg) in rats. Each bar represents the mean ± SD (n = 4 rats). * denotes statistical significance (Student’s *t*-test; *p* < 0.05).

Figure 6 reveals that all tested tissues contained measurable levels of PTX. For the control formulation (Ebetaxel), the rank order of PTX concentration (24 h post dose) from highest to lowest was kidney > lung ≈ spleen > heart ≈ liver. In contrast, for PTX micelles, the sequence was lung ≈ spleen > kidney > heart ≈ liver. Although PTX micelles had a 15% lower AUC_0-∞_ of PTX in plasma compared to Ebetaxel, the drug concentrations obtained with PTX micelles were higher in all tissues at 24 h post-dose compared to those obtained with Ebetaxel (Figure 6). However, none of the differences were found to be statistically significant except for those determined in spleen (Student’s *t*-test; *p* < 0.05) (Figure 6).

## 4. Discussion

Oral chemotherapy is still considered a challenge, and its success is expected to improve clinical practice in oncology. The use of oral chemotherapy has several advantages, which include convenience for the patient, improved quality of life, sustained drug exposure, and possible reduction in health care costs [30,31,32,33].

In recent years, a considerable amount of literature has been published on oral administration of anticancer agents. Nevertheless, most anticancer agents, especially those with superior chemotherapeutic effects such as taxanes (PTX and docetaxel), have poor oral bioavailability. The low oral bioavailability of these agents is believed to be mainly due to the first-pass metabolism by the cytochrome P450 (CYP) enzymes and the presence of plasma membrane transporter P-gp on the apical (luminal) side of enterocytes [34,35]. A growing number of preclinical and clinical studies have demonstrated that the oral bioavailability of many CYP3A and/or P-gp substrate drugs can be increased by concomitant administration of CYP3A inhibitors and/or P-gp inhibitors [36]. In fact, some pharmaceutical excipients, such as TPGS, have a demonstrated P-gp inhibiting activity both in vitro and in vivo. 

Here, we investigated the feasibility of PCL-TPGS micelles as an oral delivery system for PTX. The in vitro release study in SGF and SIF revealed that the PTX micelles had a significantly slower drug release compared to Ebetaxel. Moreover, those micelles maintained their size and PI for the entire 24 h incubation at SGF and SIF conditions, which proves that they are stable. 

We investigated the in vitro cellular uptake of PCL_7000_-TPGS_3500_ micelles in Caco-2 cells using Rh123, a well-known lipophilic P-gp fluorescent probe. The objective of this experiment was to evaluate whether loading this molecule into PCL_7000_-TPGS_3500_ micelles would enhance its permeability by inhibiting and/or bypassing P-gp. By examining the mean fluorescence intensity, we found that PCL7_000_-TPGS_3500_ micelles significantly enhanced the cellular uptake of Rh123 when compared to free Rh123. Moreover, to further study whether the presence of TPGS moiety in the copolymer was contributing to the enhancement of the permeability, we compared the results with those obtained with Rh123-loaded PCL_7000_-PEO_4000_. Indeed, the enhancement in the mean fluorescence intensity was more pronounced in the PCL_7000_-TPGS_3500_ micelles (Group III vs. Group V; Figure 3) although the difference between the two groups was statistically non-significant (*p* > 0.05; ANOVA followed by Tukey–Kramer post-hoc analysis). 

Group VI, where Rh123 was combined with TPGS_1000_ served as a positive control and to validate the experiment since TPGS_1000_ is a well-known P-gp inhibitor and absorption enhancer [37,38,39,40]. Group VII, which utilized Rh123 in combination with TPGS_3500_, which is the same TPGS variant used in the block copolymer we used to prepare PTX micelles. As expected, in comparison to free Rh123 group, TPGS_1000_ showed a significant enhancement in the cellular uptake of Rh123 in Caco-2 cells as evidenced by the fluorescence microscope image and the mean fluorescence intensity depicted in Figure 3. Additionally, the cellular uptake of Rh123 obtained in the presence of TPGS_1000_ was higher than that obtained with TPGS_3500_ (Group VII). This is in line with the findings of Collnot et al., where they synthesized TPGS analogs containing different PEG chain length (ranging from 200 to 6000) and studied their P-gp-inhibiting activities in Caco-2 monolayers [38]. They showed that while several analogues of TPGS possess P-gp-inhibiting activity, including TPGS_3500_, TPGS_1000_ showed the most potent P-gp-inhibiting activity. Interestingly, however, the intracellular mean fluorescence intensity of Rh123 obtained with TPGS_1000_ (Group VI) was comparable to both Group III (Rh123 loaded PCL_7000_-TPGS_3500_ micelles) and Group IV (Rh123 dissolved in Ebetaxel vehicle).

Since α-tocopheryl succinate moiety of PCL_7000_-TPGS_3500_ is hydrophobic, it would typically be embedded in the hydrophobic core upon micelle formation (facilitated by PEG folding). Therefore, it is reasonable to wonder how did TPGS_3500_ in the PCL_7000_-TPGS_3500_ micelles interacted with P-gp (i.e., inhibited its function) and enhanced the cellular uptake of Rh123?

We hypothesize that TPGS_3500_ can maintain its P-gp-inhibiting activity through one or more of the following mechanisms. First, micelles are dynamic structures in an equilibrium state, allowing for components to rearrange (unimers ↔ micelles). Consequently, PCL_7000_-TPGS_3500_ unimers can partially migrate to the micelle–water and micelle–cell membrane interfaces, enabling the TPGS_3500_ moiety to interact with P-gp. Second, upon interaction with the biological environment, there may be some released TPGS molecules from the micelles, either through partial micelle dissociation or PCL_7000_-TPGS_3500_ ester hydrolysis, rendering TPGS_3500_ molecules available to inhibit P-gp. Third, once intact PCL_7000_-TPGS_3500_ micelles are taken up by cells, such as Caco-2 cells and enterocytes, the micelles disassemble, liberating TPGS_3500_ to exert its action on P-gp.

One can argue that it is also possible that the enhanced uptake of Rh123 (P-gp probe) observed with PCL_7000_-TPGS_3500_ was not necessarily due to a direct effect of TPGS_3500_ moiety on the P-gp pump. Instead, it could be due to the ability of the micelles to protect the payload (i.e., Rh123 or PTX) from being recognized and pumped out by P-gp, thereby bypassing P-gp effect. However, this was unlikely to be the case for PCL_7000_-TPGS_3500_ micelles, as this bypassing effect was not observed with PCL_7000_-PEO_4000_ micelles (i.e., micelles with no tocopheryl succinate moiety). Figure 3 shows that the mean fluorescence intensity obtained with Group III (Rh123 in PCL_7000_-TPGS_3500_ micelles) was higher than that obtained with Group V (Rh123 in PCL_7000_-PEO_4000_), although the difference was statistically non-significant (*p* > 0.05). 

It is also worth noting that other research groups who worked on nanoparticles of PCL-TPGS and PLA-TPGS showed that coupling TPGS to a large molecular weight PCL or PLA did not compromise its ability to inhibit P-gp [41,42]. In other instances, conjugating TPGS to other molecules such as glycyrrhetinic acid or lactobionic acid even increased the P-gp-inhibiting activity of TPGS [43]. 

The ex vivo intestinal permeability studies depicted in Figure 4 and Table 2 provide valuable insights into the transport mechanisms of PTX across intestinal segments when delivered via PTX micelles and Ebetaxel. Initially, PTX micelles demonstrate a higher rate of permeation through non-everted intestinal sacs as compared to Ebetaxel, which aligns with the rapid absorption phase observed in the in vivo pharmacokinetic study (Figure 5). However, the permeation rates for Ebetaxel surpass those of the PTX micelles after 4 h. This suggests that while PTX micelles facilitate quicker initial uptake, Ebetaxel may offer a more sustained release, potentially leading to prolonged exposure of intestinal tissues to PTX.

The apparent permeability (*P_app_*) values and the flux (*J*) of PTX from both formulations through everted and non-everted gut sacs further support this observation. The *P_app_* values presented in Table 2 show that PTX micelles have a higher permeability ratio in everted versus non-everted sacs (2:1), indicating that these micelles may be more effective in traversing the intestinal barrier in a direction consistent with absorption into the systemic circulation. In contrast, the reduced *P_app_* ratio for Ebetaxel (1.2:1) suggests that the vehicle components, specifically Kolliphor EL (previously known as Cremophor EL), likely exerted their P-gp-inhibitory effect, thereby affecting the drug’s permeability. 

The non-significant difference in the rate of permeation and the *P_app_* values between the PTX micelles and Ebetaxel could be reflective of multiple factors, including the intrinsic properties of the micelles and the influence of the vehicle. Although the Ebetaxel vehicle might inhibit P-gp, leading to higher permeation rates through non-everted sacs, the micellar formulation of PTX seems to improve the initial uptake. This could be attributed to the micelles’ ability to solubilize and protect PTX, enhancing its diffusion through the intestinal membrane during the initial phase of absorption.

Correlating these ex vivo results with the in vitro cellular uptake and in vivo data provides a comprehensive view of the absorption process. The rapid uptake of PTX in the presence of PTX micelles corresponds to the observed initial high permeation rates. This is also in agreement with the in vivo plasma concentration–time profiles, where PTX micelles showed a rapid drug absorption reaching the C_max_ within 1 h (T_max_), whereas Ebetaxel showed a relatively slower absorption phase (T_max_ = 2 h). 

The tissue distribution study provided valuable insights into the fate of the drug in vivo 24 h post-dose. The PTX-loaded micelles showed unique distribution patterns when compared to Ebetaxel. Specifically, while the overall bioavailability of PTX in the micelles was comparable to Ebetaxel, PTX micelles consistently showed a trend of higher concentrations of PTX in all the studied tissues in comparison to Ebetaxel. Nonetheless, the difference in concentrations reached statistical significance (*p* < 0.05; Student’s *t*-test) only in the spleen tissue. This may suggest a different absorption pathway for PTX micelles (e.g., lymphatic system) or that PTX micelles were detected by the reticuloendothelial system. 

The sustained in vitro release profile of PTX from the micelles in SGF and SIF suggests that the micelles may have acted as a depot for PTX in the GI tract. The slow and sustained release of PTX from the micelles over time indicates that the drug was continuously being released from the formulation, potentially contributing to the observed higher tissue concentrations. The higher drug concentrations in the tissues observed with PTX micelles compared to Ebetaxel^®^ despite the undetectable concentrations of the drug in plasma beyond 8 h post-dose in the PTX micelle group further support the hypothesis that the micelles acted as a reservoir, continuously releasing PTX over an extended period. However, it should be noted that the tissue distribution profile here was based on a single time point (24 h post-dose). 

In order to draw a definitive conclusion whether or not PTX micelles resulted in a tissue distribution profile that is significantly different from the control formulation, the biodistribution study should be designed differently. The design of such experiment should allow for organs to be collected at multiple time intervals to facilitate the calculation of AUC of drug in each tissue. Because AUC is a better index for calculation of drug exposure rather than an average tissue concentration obtained at a single time point. 

Nonetheless, taken together, the findings from this study indicate that the PCL_7000_-TPGS_3500_ micellar formulation has the potential to significantly improve the oral absorption of PTX and enhance its intestinal permeability. In fact, with PTX serving as a model drug, the results suggest that these micelles can potentially improve the oral absorption/bioavailability of any lipophilic drug that is in BCS Class II–IV (low solubility and/or permeability). 

The shift from IV to oral administration of chemotherapeutic agents could notably improve patient quality of life by reducing the need for frequent hospital visits, lowering healthcare costs, and potentially diminishing severe systemic side effects associated with high peak plasma concentrations typical of IV administration [33]. Further preclinical and clinical investigations are necessary to confirm these promising results and to fully establish the clinical safety and efficacy profile of this micellar formulation. Specifically, the next steps should include studies to investigate the pharmacokinetic/tissue distribution profile as well as the safety and efficacy of the developed formulation in tumor-bearing animals. 

Ensuring the quality of the final product through the implementation and validation of good manufacturing practices (GMPs) is crucial for the clinical translation of drug formulations, including polymeric micellar formulations. However, many production processes for polymeric micelles face issues with reproducibility and scalability, making it challenging to meet GMP standards.

In this study, a co-solvent evaporation method was used to produce micelles. Compared to dialysis, another widely used method, the co-solvent evaporation method offers several advantages, including better scalability and a reduced risk of drug loss during the encapsulation process.

Nevertheless, future studies should explore the feasibility of utilizing modern technologies such as microfluidic systems for large-scale production of polymeric micelles. Microfluidic mixing-based production methods can provide superior control in achieving the desired size, morphology, shape, size distribution, and surface properties of the prepared polymeric micelles [44,45,46].

## 5. Conclusions

The PCL_7000_-TPGS_3500_ micellar formulation presents a promising alternative for the oral delivery of paclitaxel, addressing significant challenges associated with its solubility and bioavailability. The micelles not only improved the solubility and stability of PTX but also demonstrated potential for enhanced intestinal absorption and sustained drug release. These results indicate that PTX-loaded PCL-TPGS micelles may serve as an effective formulation for oral delivery of the drug. However, additional preclinical and clinical studies are needed to confirm these findings.

## Figures and Tables

**Table 1:** Characteristics of the polymeric micellar formulation of PTX.

Block Copolymer	Mn (g/mol) ^a^	Size (nm) ^b^	PI ^c^	Encapsulation Efficiency ± SD (%) ^d^
PCL_7000_-TPGS_3500_	10,400	67.73 ± 0.46	0.293 ± 0.055	92.75 ± 2.25

^a^ Number-average molecular weight of the copolymer measured by ^1^H NMR. ^b^ Mean size estimated by DLS technique. ^c^ Polydispersity (PI) measured by DLS technique. ^d^ Measured by HPLC. Values are recorded as mean ± SD (n = 4–6).

**Table 2:** Apparent permeability (*P_app_*) and flux (*J*) of PTX from PTX micelles and Ebetaxel through everted and non-everted gut sacs.

Formulation	*P_app_* × 10^−5^ (cm/min)	Flux (*J*) (ng/cm^2^·min)
PTX micelles (non-everted)	1.16 ± 0.35	2.4 ± 0.6
PTX micelles (everted)	2.34 ± 0.63	4.7 ± 1.2
Ebetaxel (non-everted)	1.94 ± 0.47	3.9 ± 0.7
Ebetaxel (everted)	2.43 ± 1.36	4.8 ± 2.7

Data were represented as mean ± SD (n = 5/group).

**Table 3:** Pharmacokinetics of PTX in plasma after oral administration of Ebetaxel or PTX-loaded micelles to rats at a dose of 10 mg/kg.

Parameter	Ebetaxel^®^	PTX-Loaded Micelles
AUC_0-tlast_ (ng·h/mL)	303.95 ± 36.34	249.94 ± 105.86
AUC_0-∞_ (ng·h/mL)	375.87 ± 93.72	317.03 ± 173.12
t_1/2_ (h)	4.71 ± 1.93	3.12 ± 1.47
CL/F (L/kg/h)	28.40 ± 7.25	39.23 ± 14.64
C_max_ (ng/mL)	59.60 ± 8.17	96.62 ± 33.74
T_max_ (h) ^a^	2	1

^a^ Data were presented as median. AUC_0-tlast_: AUC from time zero to the last measurable concentration. Data were represented as mean ± SD (n = 4/group).

## Data Availability

The original contributions presented in the study are included in the article, further inquiries can be directed to the corresponding author.

## Supplementary Materials

The following supporting information can be downloaded at: https://www.mdpi.com/article/10.3390/polym16152232/s1, Figure S1: Full size images presented in Figure 3.

## References

[1] Piccart-Gebhart M.J., Burzykowski T., Buyse M., Sledge G., Carmichael J., Lück H.-J., Mackey J.R., Nabholtz J.-M., Paridaens R., Biganzoli L. (2008). Taxanes Alone or in Combination With Anthracyclines As First-Line Therapy of Patients With Metastatic Breast Cancer. J. Clin. Oncol..

[2] Sparano J.A., Wang M., Martino S., Jones V., Perez E.A., Saphner T., Wolff A.C., Sledge G.W., Wood W.C., Davidson N.E. (2008). Weekly Paclitaxel in the Adjuvant Treatment of Breast Cancer. N. Engl. J. Med..

[3] Homesley H.D., Filiaci V., Markman M., Bitterman P., Eaton L., Kilgore L.C., Monk B.J., Ueland F.R. (2007). Phase III Trial of Ifosfamide With or Without Paclitaxel in Advanced Uterine Carcinosarcoma: A Gynecologic Oncology Group Study. J. Clin. Oncol..

[4] Gill P.S., Tulpule A., Espina B.M., Cabriales S., Bresnahan J., Ilaw M., Louie S., Gustafson N.F., Brown M.A., Orcutt C. (1999). Paclitaxel Is Safe and Effective in the Treatment of Advanced AIDS-Related Kaposi’s Sarcoma. J. Clin. Oncol..

[5] Kumar S., Mahdi H., Bryant C., Shah J.P., Garg G., Munkarah A. (2010). Clinical Trials and Progress with Paclitaxel in Ovarian Cancer. Int. J. Women’s Health.

[6] Hoskins P.J., Swenerton K.D., Pike J.A., Wong F., Lim P., Acquino-Parsons C., Lee N. (2001). Paclitaxel and Carboplatin, Alone or With Irradiation, in Advanced or Recurrent Endometrial Cancer: A Phase II Study. J. Clin. Oncol..

[7] Paz-Ares L., Bálint B., De Boer R.H., Van Meerbeeck J.P., Wierzbicki R., De Souza P., Galimi F., Haddad V., Sabin T., Hei Y. (2013). A Randomized Phase 2 Study of Paclitaxel and Carboplatin with or without Conatumumab for First-Line Treatment of Advanced Non–Small-Cell Lung Cancer. J. Thorac. Oncol..

[8] Kumar G., Mullick P., Nandakumar K., Mutalik S., Rao C.M. (2022). Box–Behnken Design-Based Development and Validation of a Reverse-Phase HPLC Analytical Method for the Estimation of Paclitaxel in Cationic Liposomes. Chromatographia.

[9] Sofias A.M., Dunne M., Storm G., Allen C. (2017). The Battle of “Nano” Paclitaxel. Adv. Drug Deliv. Rev..

[10] Meerum Terwogt J.M., Malingré M.M., Beijnen J.H., ten Bokkel Huinink W.W., Rosing H., Koopman F.J., van Tellingen O., Swart M., Schellens J.H. (1999). Coadministration of Oral Cyclosporin A Enables Oral Therapy with Paclitaxel. Clin. Cancer Res..

[11] Sparreboom A., Van Asperen J., Mayer U., Schinkel A.H., Smit J.W., Meijer D.K.F., Borst P., Nooijen W.J., Beijnen J.H., Van Tellingen O. (1997). Limited Oral Bioavailability and Active Epithelial Excretion of Paclitaxel (Taxol) Caused by P-Glycoprotein in the Intestine. Proc. Natl. Acad. Sci. USA.

[12] Chu Z., Chen J.-S., Liau C.-T., Wang H.-M., Lin Y.-C., Yang M.-H., Chen P.-M., Gardner E.R., Figg W.D., Sparreboom A. (2008). Oral Bioavailability of a Novel Paclitaxel Formulation (Genetaxyl) Administered with Cyclosporin A in Cancer Patients. Anti-Cancer Drugs.

[13] Veltkamp S.A., Rosing H., Huitema A.D.R., Fetell M.R., Nol A., Beijnen J.H., Schellens J.H.M. (2007). Novel Paclitaxel Formulations for Oral Application: A Phase I Pharmacokinetic Study in Patients with Solid Tumours. Cancer Chemother. Pharmacol..

[14] Helgason H.H., Kruijtzer C.M.F., Huitema A.D.R., Marcus S.G., Ten Bokkel Huinink W.W., Schot M.E., Schornagel J.H., Beijnen J.H., Schellens J.H.M. (2006). Phase II and Pharmacological Study of Oral Paclitaxel (Paxoral) plus Ciclosporin in Anthracycline-Pretreated Metastatic Breast Cancer. Br. J. Cancer.

[15] Kruijtzer C.M.F., Schellens J.H.M., Mezger J., Scheulen M.E., Keilholz U., Beijnen J.H., Rosing H., Mathôt R.A.A., Marcus S., Van Tinteren H. (2002). Phase II and Pharmacologic Study of Weekly Oral Paclitaxel Plus Cyclosporine in Patients With Advanced Non–Small-Cell Lung Cancer. J. Clin. Oncol..

[16] Malingré M.M., Terwogt J.M.M., Beijnen J.H., Rosing H., Koopman F.J., Van Tellingen O., Duchin K., Huinink W.W.T.B., Swart M., Lieverst J. (2000). Phase I and Pharmacokinetic Study of Oral Paclitaxel. J. Clin. Oncol..

[17] Binkhathlan Z., Lavasanifar A. (2013). P-Glycoprotein Inhibition as a Therapeutic Approach for Overcoming Multidrug Resistance in Cancer: Current Status and Future Perspectives. Curr. Cancer Drug Targets.

[18] Varma M.V.S., Panchagnula R. (2005). Enhanced Oral Paclitaxel Absorption with Vitamin E-TPGS: Effect on Solubility and Permeability in Vitro, in Situ and in Vivo. Eur. J. Pharm. Sci..

[19] Yusuf O., Ali R., Alomrani A.H., Alshamsan A., Alshememry A.K., Almalik A.M., Lavasanifar A., Binkhathlan Z. (2021). Design and Development of D–α–Tocopheryl Polyethylene Glycol Succinate–block–Poly(ε-Caprolactone) (TPGS−b−PCL) Nanocarriers for Solubilization and Controlled Release of Paclitaxel. Molecules.

[20] Bilensoy E., Gürkaynak O., Ertan M., Şen M., Hıncal A.A. (2008). Development of Nonsurfactant Cyclodextrin Nanoparticles Loaded With Anticancer Drug Paclitaxel. J. Pharm. Sci..

[21] Guo S., Pham K., Li D., Penzak S., Dong X. (2016). Novel in Situ Self-Assembly Nanoparticles for Formulating a Poorly Water-Soluble Drug in Oral Solid Granules, Improving Stability, Palatability, and Bioavailability. Int. J. Nanomed..

[22] Moore J.W., Flanner H.H. (1996). Mathematical Comparison of Dissolution Profiles. Pharm. Technol..

[23] Costa P., Sousa Lobo J.M. (2001). Modeling and Comparison of Dissolution Profiles. Eur. J. Pharm. Sci..

[24] Jhala D., Rather H., Kedaria D., Shah J., Singh S., Vasita R. (2019). Biomimetic Polycaprolactone-Chitosan Nanofibrous Substrate Influenced Cell Cycle and ECM Secretion Affect Cellular Uptake of Nanoclusters. Bioact. Mater..

[25] Ibrahim W., Al-Omrani A., Yassin A.E. (2013). Novel Sulpiride-Loaded Solid Lipid Nanoparticles with Enhanced Intestinal Permeability. Int. J. Nanomed..

[26] Mateer S.W., Cardona J., Marks E., Goggin B.J., Hua S., Keely S. (2016). Ex Vivo Intestinal Sacs to Assess Mucosal Permeability in Models of Gastrointestinal Disease. J. Vis. Exp..

[27] Sánchez A.B., Calpena A.C., Mallandrich M., Clares B. (2019). Validation of an Ex Vivo Permeation Method for the Intestinal Permeability of Different BCS Drugs and Its Correlation with Caco-2 In Vitro Experiments. Pharmaceutics.

[28] Narade S., Pore Y. (2019). Optimization of Ex Vivo Permeability Characteristics of Berberine in Presence of Quercetin Using 32 Full Factorial Design. J. App Pharm. Sci..

[29] Binkhathlan Z., Yusuf O., Ali R., Alomrani A.H., Alshamsan A., Alshememry A.K., Almomen A., Alkholief M., Aljuffali I.A., Alqahtani F. (2024). Polycaprolactone—Vitamin E TPGS Micelles for Delivery of Paclitaxel: In Vitro and in Vivo Evaluation. Int. J. Pharm. X.

[30] Aisner J. (2007). Overview of the Changing Paradigm in Cancer Treatment: Oral Chemotherapy. Am. J. Health-Syst. Pharm..

[31] Weingart S.N., Brown E., Bach P.B., Eng K., Johnson S.A., Kuzel T.M., Langbaum T.S., Leedy R.D., Muller R.J., Newcomer L.N. (2008). NCCN Task Force Report: Oral Chemotherapy. J. Natl. Compr. Canc Netw..

[32] Liu G., Franssen E., Fitch M.I., Warner E. (1997). Patient Preferences for Oral versus Intravenous Palliative Chemotherapy. J. Clin. Oncol..

[33] Schoener C.A., Peppas N.A. (2012). Oral Delivery of Chemotherapeutic Agents: Background and Potential of Drug Delivery Systems for Colon Delivery. J. Drug Deliv. Sci. Technol..

[34] Stuurman F.E., Nuijen B., Beijnen J.H., Schellens J.H.M. (2013). Oral Anticancer Drugs: Mechanisms of Low Bioavailability and Strategies for Improvement. Clin. Pharmacokinet..

[35] Eisenmann E.D., Talebi Z., Sparreboom A., Baker S.D. (2022). Boosting the Oral Bioavailability of Anticancer Drugs through Intentional Drug–Drug Interactions. Basic. Clin. Pharma Tox.

[36] Zhang Y., Benet L.Z. (2001). The Gut as a Barrier to Drug Absorption: Combined Role of Cytochrome P450 3A and P-Glycoprotein. Clin. Pharmacokinet..

[37] Collnot E.-M., Baldes C., Schaefer U.F., Edgar K.J., Wempe M.F., Lehr C.-M. (2010). Vitamin E TPGS P-Glycoprotein Inhibition Mechanism: Influence on Conformational Flexibility, Intracellular ATP Levels, and Role of Time and Site of Access. Mol. Pharm..

[38] Collnot E.-M., Baldes C., Wempe M.F., Hyatt J., Navarro L., Edgar K.J., Schaefer U.F., Lehr C.-M. (2006). Influence of Vitamin E TPGS Poly(Ethylene Glycol) Chain Length on Apical Efflux Transporters in Caco-2 Cell Monolayers. J. Control. Release.

[39] Collnot E.-M., Baldes C., Wempe M.F., Kappl R., Hüttermann J., Hyatt J.A., Edgar K.J., Schaefer U.F., Lehr C.-M. (2007). Mechanism of Inhibition of P-Glycoprotein Mediated Efflux by Vitamin E TPGS: Influence on ATPase Activity and Membrane Fluidity. Mol. Pharm..

[40] Tavares Luiz M., Delello Di Filippo L., Carolina Alves R., Sousa Araújo V.H., Lobato Duarte J., Maldonado Marchetti J., Chorilli M. (2021). The Use of TPGS in Drug Delivery Systems to Overcome Biological Barriers. Eur. Polym. J..

[41] Zhang Z., Feng S. (2006). Self-Assembled Nanoparticles of Poly(Lactide)–Vitamin E TPGS Copolymers for Oral Chemotherapy. Int. J. Pharm..

[42] Bernabeu E., Gonzalez L., Legaspi M.J., Moretton M.A., Chiappetta D.A. (2016). Paclitaxel-Loaded TPGS-*b*-PCL Nanoparticles: In Vitro Cytotoxicity and Cellular Uptake in MCF-7 and MDA-MB-231 Cells versus mPEG-*b*-PCL Nanoparticles and Abraxane ^®^. J. Nanosci. Nanotechnol..

[43] Liu T., Liu X., Xiong H., Xu C., Yao J., Zhu X., Zhou J., Yao J. (2018). Mechanisms of TPGS and Its Derivatives Inhibiting P-Glycoprotein Efflux Pump and Application for Reversing Multidrug Resistance in Hepatocellular Carcinoma. Polym. Chem..

[44] Karnik R., Gu F., Basto P., Cannizzaro C., Dean L., Kyei-Manu W., Langer R., Farokhzad O.C. (2008). Microfluidic Platform for Controlled Synthesis of Polymeric Nanoparticles. Nano Lett..

[45] Capretto L., Mazzitelli S., Brognara E., Lampronti I., Carugo D., Hill M., Zhang X., Gambari R. (2012). Gambari Mithramycin Encapsulated in Polymeric Micelles by Microfluidic Technology as Novel Therapeutic Protocol for Beta-Thalassemia. Int. J. Nanomed..

[46] Ahmadi M., Siavashy S., Ayyoubzadeh S.M., Kecili R., Ghorbani-Bidkorbeh F. (2021). Controllable Synthesis of Polymeric Micelles by Microfluidic Platforms for Biomedical Applications: A Systematic Review. Iran. J. Pharm. Res..

