# Supplementary material for: Polycaprolactone—Vitamin E TPGS Micellar Formulation for Oral Delivery of Paclitaxel
Source: Polymers (Basel). 2024 Aug 5;16(15):2232. doi: 10.3390/polym16152232 (PMC11314731; doi:10.3390/polym16152232)

Supplementary Materials

Polycaprolactone—Vitamin E TPGS Micellar Formulation for Oral Delivery of Paclitaxel

Figure S1. Full size images presented in Figure 3A:

Group I: Negative control

Bright field

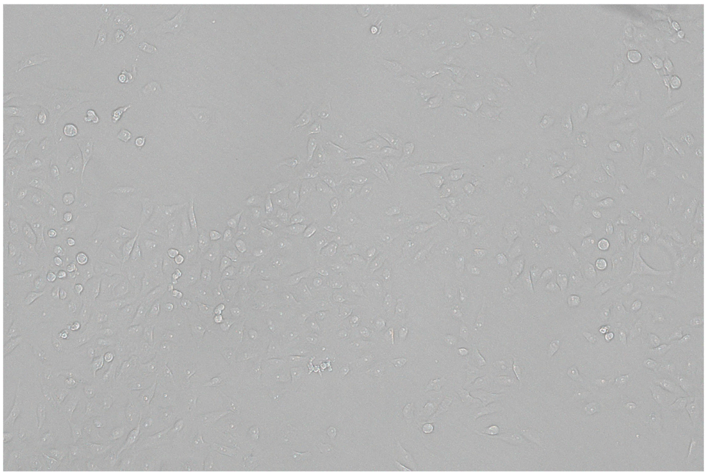

Fluorescent

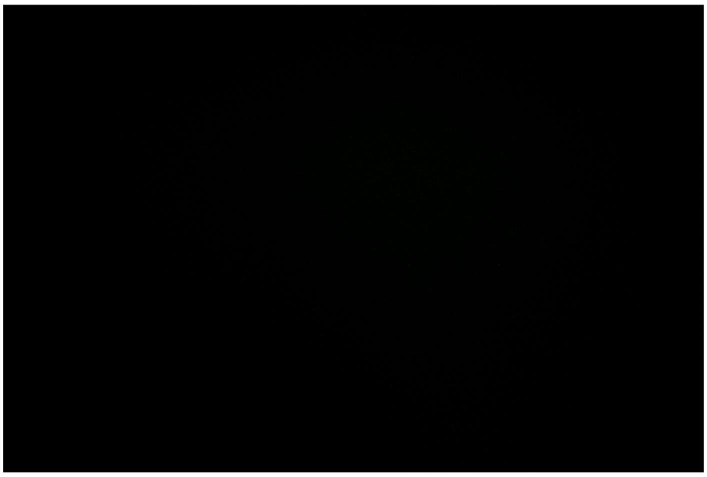

Merged

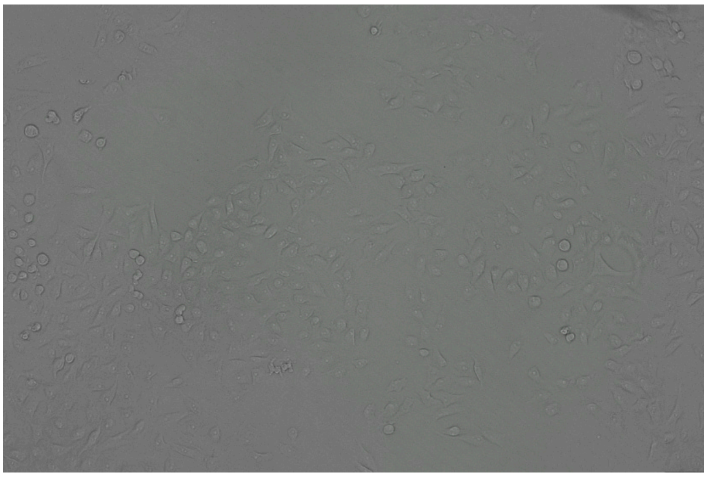

Group II: Free Rh123 (20μM)

Bright field

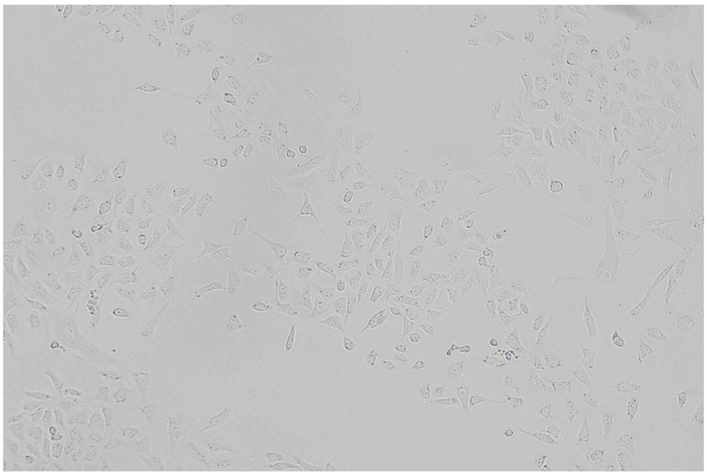

Fluorescent

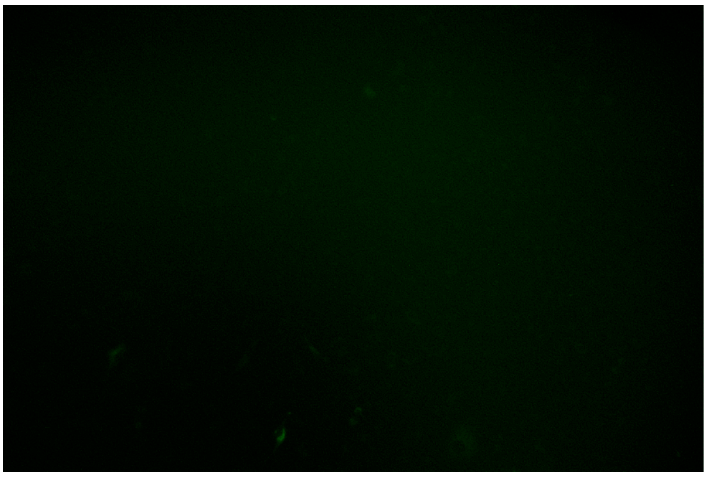

Merged

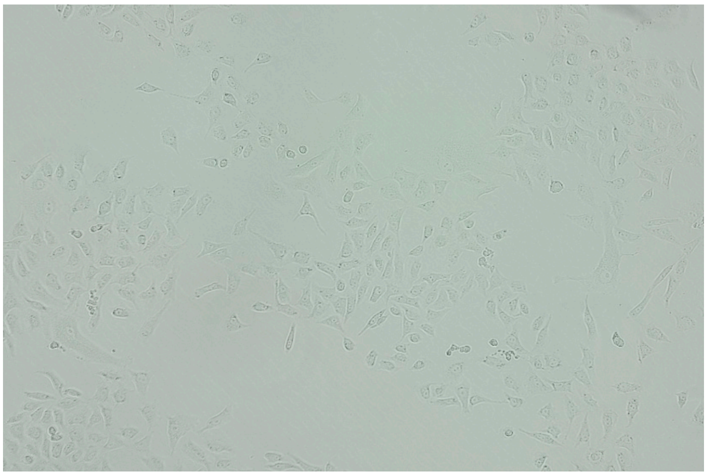

Group III: Rh123 (20μM) encapsulated in PCL_{7000}-$TPGS_{3500}$ micelles

Bright field

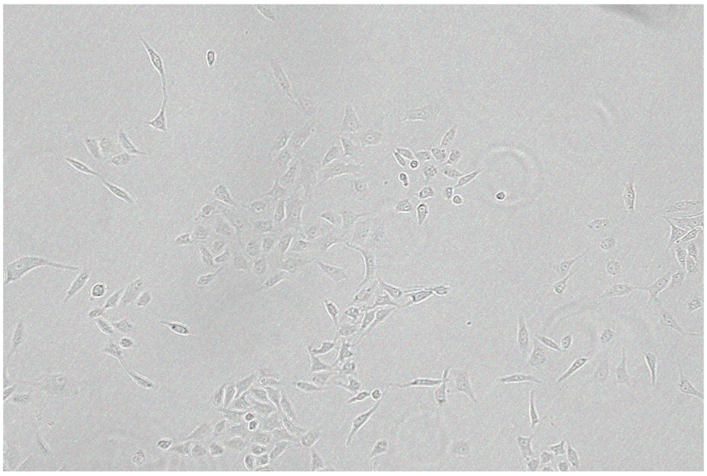

Fluorescent

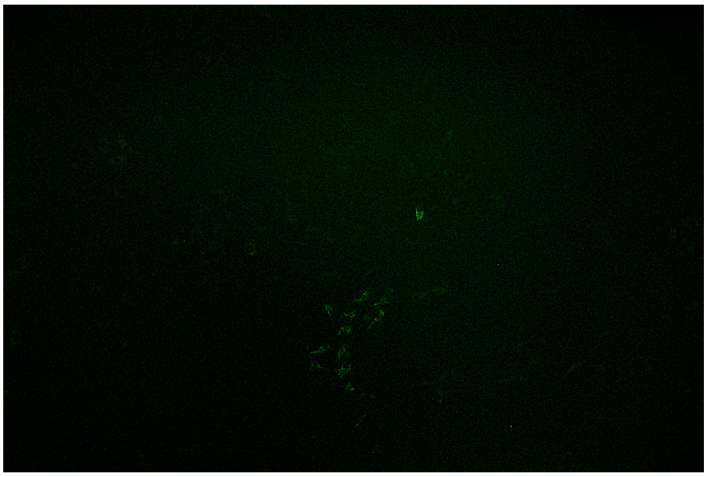

Merged

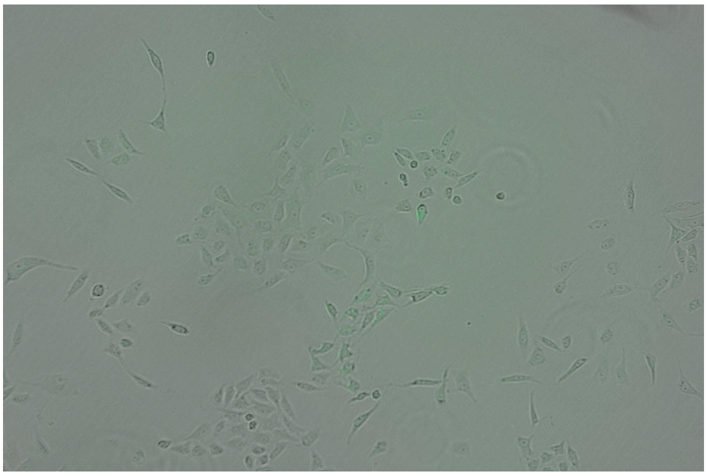

Group IV: Rh123 (20μM) in Ebetaxel vehicle (mixture of Kolliphor EL and ethanol

Bright field

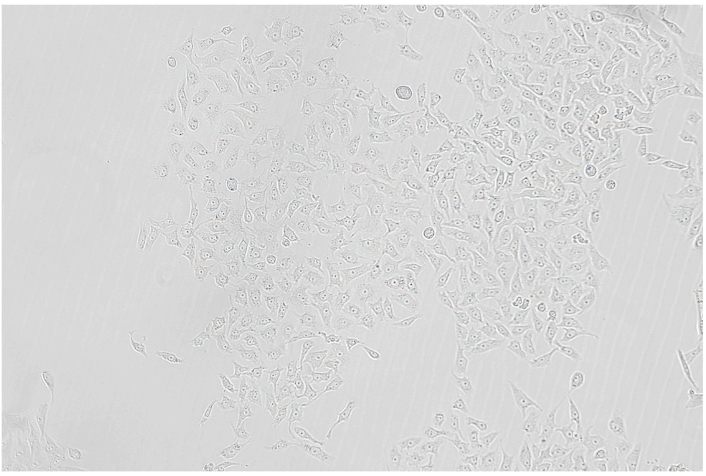

Fluorescent

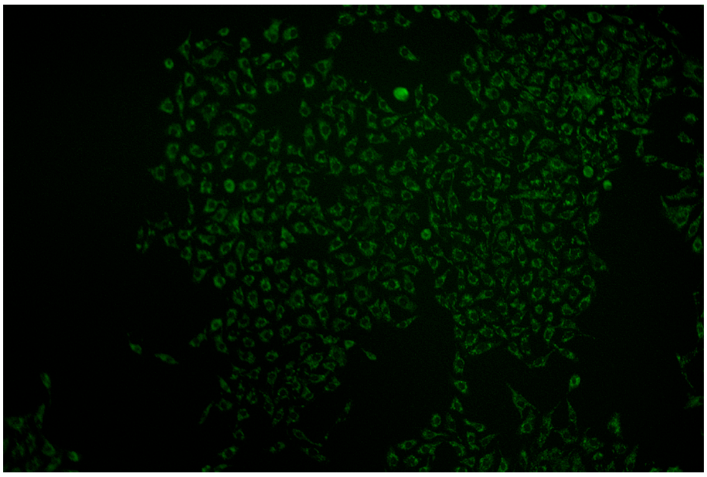

Merged

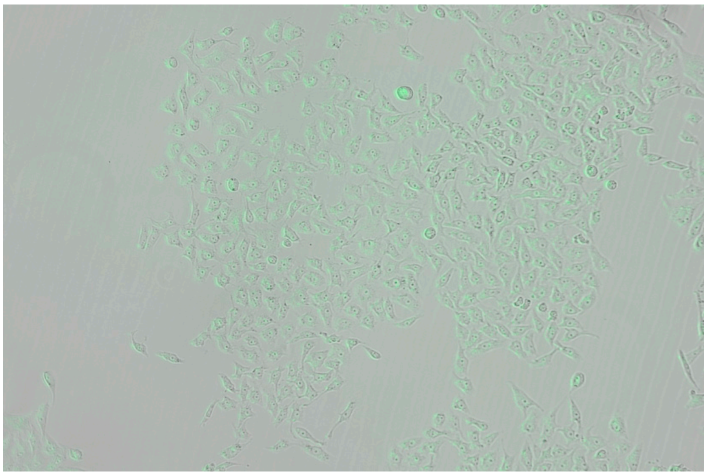

Group V: Rh123 (20μM) encapsulated in PEO_{4000}-PCL_{7000} micelles

Bright field

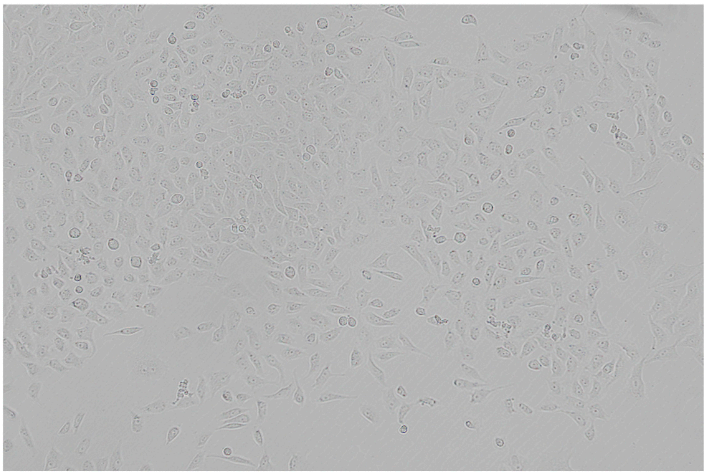

Fluorescent

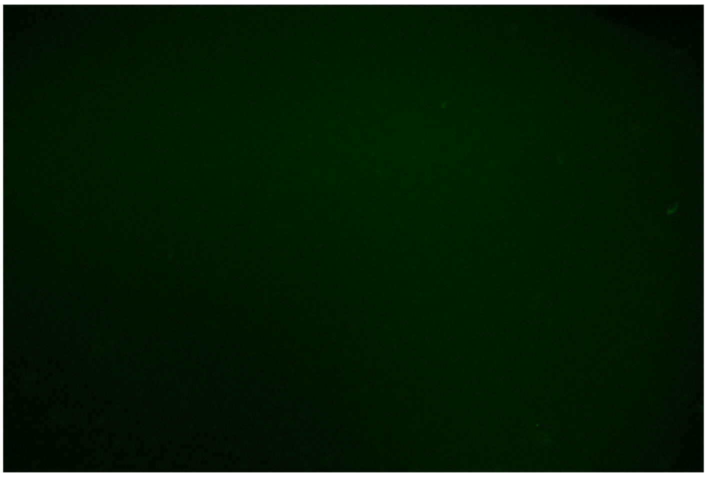

Merged

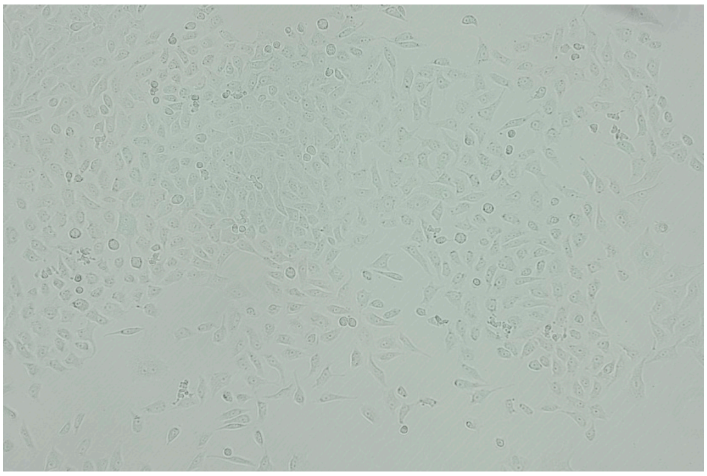

Group VI: Solution mixture of Rh123 (20μM) and $TPGS_{1000}$ (33μM)

Bright field

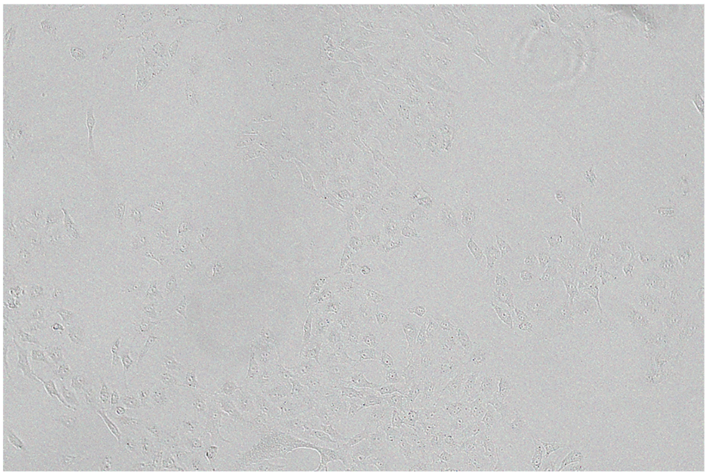

Fluorescent

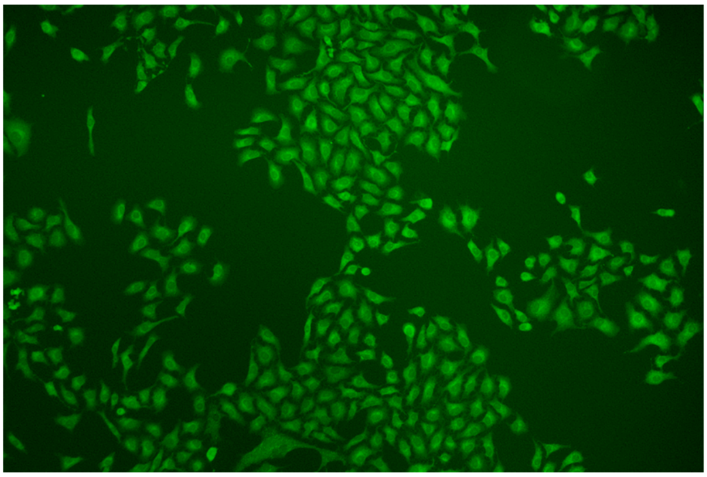

Merged

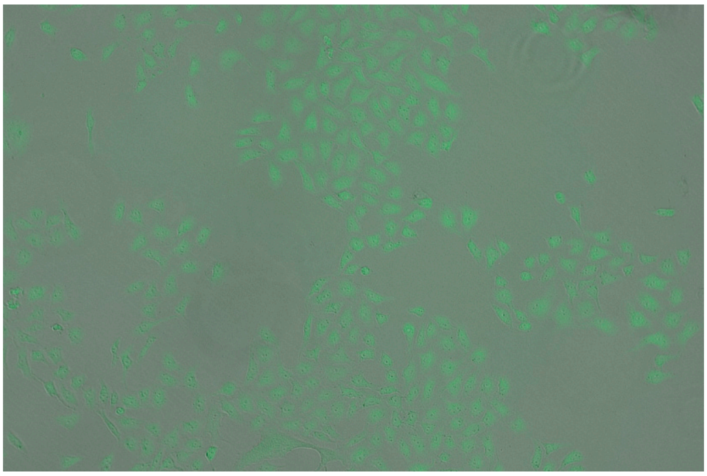

Group VII: Solution mixture of Rh123 (20μM) and $TPGS_{3500}$ (33μM)

Bright field

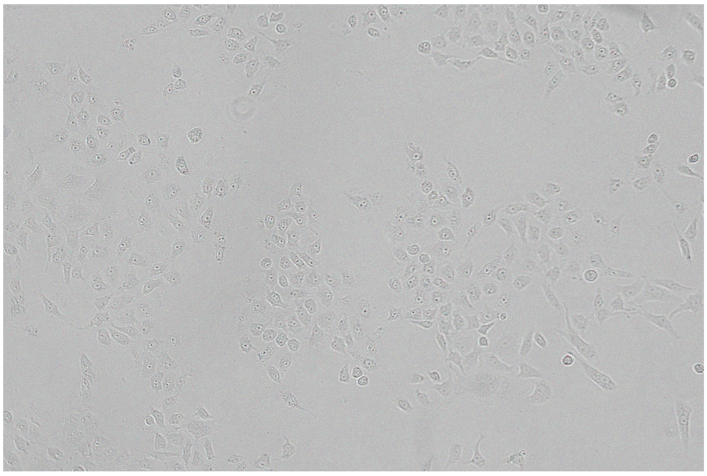

Fluorescent

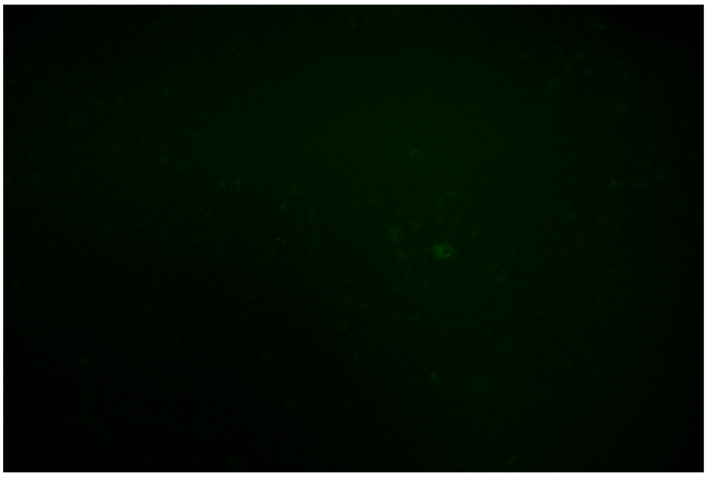

Merged

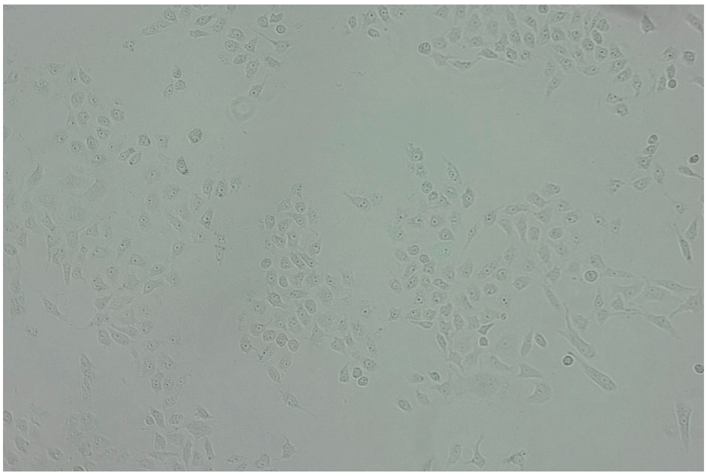

Group VIII: Solution mixture of Rh123 (20μM) and Cyclosporine A (4μM)

Bright field

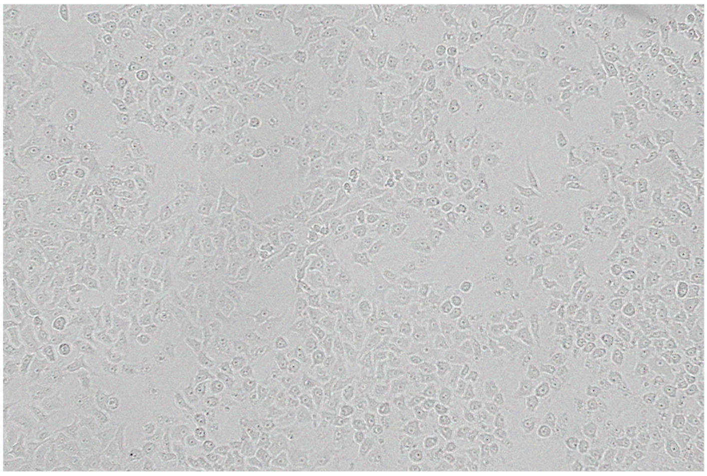

Fluorescent

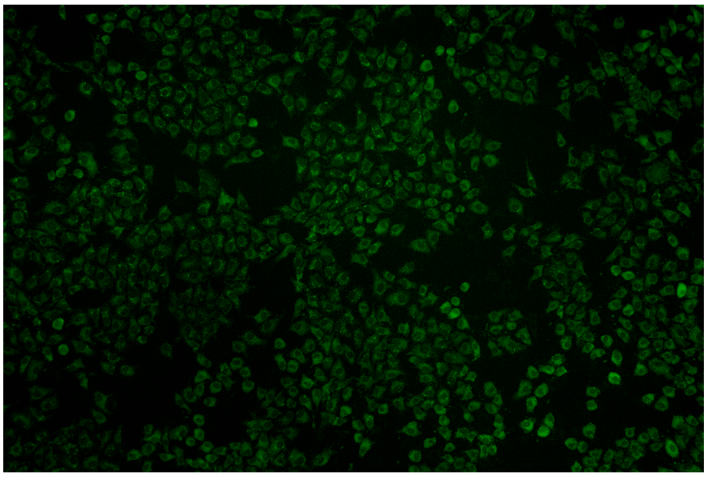

Merged

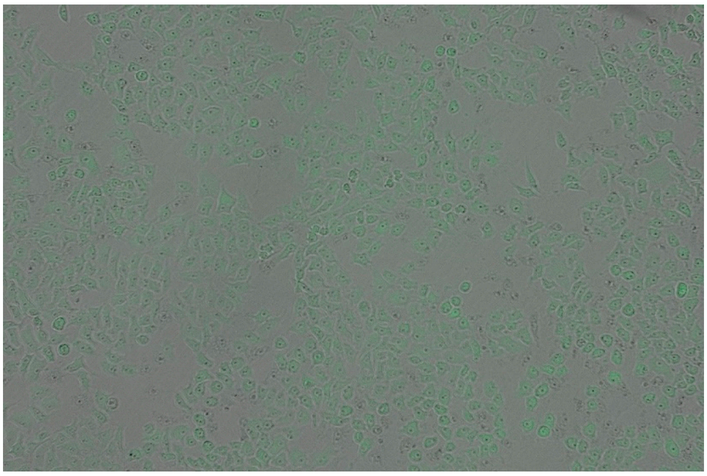

Supplement: Supplementary file 1 [file polymers-16-02232-s001.zip › polymers-3097572-supplementary.pdf]
